# Supplementary material for: Digital health literacy, online information-seeking behaviour, and satisfaction of Covid-19 information among the university students of East and South-East Asia
Source: PLoS One. 2022 Apr 13;17(4):e0266276. doi: 10.1371/journal.pone.0266276 (PMC9007389; doi:10.1371/journal.pone.0266276)
Supplement: S1 Table — (DOCX) [file pone.0266276.s001.docx]

**Supporting information**

S1 Table. Digital health literacy (n=4,890)*

| **DHL Scale** | **All** | **China** | **Philippines** | **Malaysia** |
| --- | --- | --- | --- | --- |
|  | Mean (SD) | Mean (SD) | Mean (SD) | Mean (SD) |
| **Overall digital health literacy score**  *(excluding subscale 5)* | **2.89 (0.42)** | **2.89 (0.43)** | **2.88 (0.43)** | **2.93 (0.40)** |
| **Subscale 1: Information Seeking**   1. …make a choice from all the information you find 2. …use the proper words or search query to find the information you are looking for 3. …find the exact information you are looking for | **3.01 (0.51)**  2.92 (0.63)  3.12 (0.57)  2.99 (0.64) | **3.05 (0.51)**  2.96 (0.62)  3.13 (0.56)  3.06 (0.58) | **2.94 (0.51)**    2.84 (0.64)  3.10 (0.57)  2.90 (0.67) | **3.09 (0.49)**  3.03 (0.58)  3.19 (0.56)  3.04 (0.62) |
| **Subscale 2: Adding self-generated content**   1. …clearly formulate your question or health-related worry 2. …express your opinion, thoughts, or feelings in writing 3. …write your message as such, for people to understand exactly what you mean | **2.76 (0.60)**  2.83 (0.64)  2.77 (0.71)  2.71 (0.71) | **2.86 (0.61)**  2.90 (0.65)  2.87 (0.66)  2.82 (0.69) | **2.68 (0.59)**  2.76 (0.64)  2.69 (0.75)  2.61 (0.72) | **2.76 (0.56)**  2.83 (0.60)  2.75 (0.70)  2.72 (0.66) |
| **Subscale 3: Evaluating reliability**   1. …decide whether the information is reliable or not 2. decide whether the information is written with commercial interests 3. …check different websites to see whether they provide the same information | **2.78 (0.58)**  2.69 (0.73)  2.75 (0.73)  2.91 (0.66) | **2.67 (0.57)**  2.62 (0.57)  2.62 (0.71)  2.79 (0.64) | **2.86 (0.59)**  2.76 (0.75)  2.83 (0.72)  2.99 (0.67) | **2.83 (0.56)**  2.68 (0.74)  2.81 (0.77)  3.00 (0.61) |
| **Subscale 4: Determining relevance**   1. …decide if the information you found is applicable to you 2. …apply the information you found in your daily life 3. …use the information you found to make decisions about your health | **3.00 (0.48)**  2.95 (0.57)  2.96 (0.57)  3.09 (0.57) | **2.98 (0.47)**  2.92 (0.55)  2.98 (0.54)  3.04 (0.55) | **3.01 (0.49)**  2.96 (0.60)  2.95 (0.60)  3.13 (0.58) | **3.01 (0.45)**  2.96 (0.56)  2.97 (0.57)  3.11 (0.56) |
| **Subscale 5: Protecting privacy**   1. do you find it difficult to judge who can read along? 2. do you (intentionally or unintentionally) share your own private information (eg, name or address)? 3. do you (intentionally or unintentionally) share some else’s private information? | **3.12 (0.72)**  2.47 (0.98)  3.26 (0.97)  3.60 (0.79) | **3.16 (0.78)**  2.69 (1.04)  3.33 (0.95)  3.45 (0.92) | **3.06 (0.67)**  2.29 (0.90)  3.17 (0.98)  3.69 (0.69) | **3.24 (0.66)**  2.48 (0.97)  3.35 (0.96)  3.74 (0.64) |

*Excluded sample who reported “No” in online health seeking information/missing values (7.8%, n=412)
