# Supplementary material for: Digital health literacy, online information-seeking behaviour, and satisfaction of Covid-19 information among the university students of East and South-East Asia
Source: PLoS One. 2022 Apr 13;17(4):e0266276. doi: 10.1371/journal.pone.0266276 (PMC9007389; doi:10.1371/journal.pone.0266276)
Supplement: S2 Table — (DOCX) [file pone.0266276.s002.docx]

**Supporting information**

S2 Table. Importance of internet information search (n=4,890)*

|  | **All countries** | **China** | **Philippines** | **Malaysia** |
| --- | --- | --- | --- | --- |
| **Importance** | Mean (SD) | Mean (SD) | Mean (SD) | Mean (SD) |
| ***Overall importance*** | **3.62 (0.39)** | **3.43 (0.43)** | **3.80 (0.25)** | **3.57 (0.38)** |
| A. the information is up to date? | 3.76 (0.47) | 3.54 (0.56) | 3.93 (0.27) | 3.81 (0.44) |
| B. the information is verified? | 3.85 (0.40) | 3.74 (0.49) | 3.95 (0.22) | 3.82 (0.43) |
| C. you quickly learn the most important things? | 3.55 (0.59) | 3.27 (0.63) | 3.82 (0.40) | 3.50 (0.59) |
| D. the information comes from official sources? | 3.74 (0.54) | 3.53 (0.69) | 3.94 (0.26) | 3.73 (0.52) |
| E. different opinions are represented? | 3.24 (0.71) | 3.12 (0.67) | 3.41 (0.68) | 3.07 (0.76) |
| F. the subject is dealt with comprehensively? | 3.57 (0.59) | 3.37 (0.64) | 3.78 (0.45) | 3.47 (0.61) |

*Excluded sample who reported “No” in online health seeking information/missing values (7.8%, n=412)
