# Supplementary material for: Digital health literacy, online information-seeking behaviour, and satisfaction of Covid-19 information among the university students of East and South-East Asia
Source: PLoS One. 2022 Apr 13;17(4):e0266276. doi: 10.1371/journal.pone.0266276 (PMC9007389; doi:10.1371/journal.pone.0266276)
Supplement: S1 File — (DOCX) [file pone.0266276.s003.docx]

**Minimal underlying data set: Digital health literacy, online information-seeking behaviour, and satisfaction of Covid-19 information among the university students of East and South-East Asia**

| **Table 3** | Mean | SD | SE | N | Statistical method used | p-value | | | |
| --- | --- | --- | --- | --- | --- | --- | --- | --- | --- |
| Country: China |  |  |  |  |  | Info_webpublic | Info_news | Info_webdocs.ic | Info_healthports |
| DHL_search | 3.05 | 0.51 | 0.012 | 1884 | Binary logistic regression | 0.413 | 0.542 | 0.984 | 0.626 |
| DHL_content | 2.86 | 0.61 | 0.014 | 1799 |  | 0.497 | 0.018 | 0.012 | 0.772 |
| DHL_reliability | 2.67 | 0.57 | 0.013 | 1884 |  | 0.001 | 0.066 | 0.000 | 0.000 |
| DHL_relevance | 2.98 | 0.47 | 0.011 | 1883 |  | 0.010 | 0.000 | 0.425 | 0.052 |
| DHL_privacy | 3.16 | 0.78 | 0.019 | 1725 |  | 0.046 | 0.000 | 0.001 | 0.207 |
| Info_webpublic  Low use  High use |  |  |  | 751  1130 |  |  |  |  |  |
| Info_news  Low use  High use |  |  |  | 439  1442 |  |  |  |  |  |
| Info_webdocs.ic  Low use  High use |  |  |  | 1405  474 |  |  |  |  |  |
| Info_healthports  Low use  High use |  |  |  | 1189  690 |  |  |  |  |  |
| Country: Philippines | | | | | | | | | |
| DHL_search | 2.94 | 0.51 | 0.011 | 2161 | Binary logistic regression | 0.216 | 0.350 | 0.038 | 0.082 |
| DHL_content | 2.68 | 0.59 | 0.013 | 2085 |  | 0.077 | 0.204 | 0.000 | 0.000 |
| DHL_reliability | 2.86 | 0.59 | 0.013 | 2155 |  | 0.000 | 0.534 | 0.814 | 0.778 |
| DHL_relevance | 3.01 | 0.49 | 0.011 | 2154 |  | 0.577 | 0.136 | 0.457 | 0.911 |
| DHL_privacy | 3.06 | 0.67 | 0.015 | 2043 |  | 0.231 | 0.514 | 0.006 | 0.252 |
| Info_webpublic  Low use  High use |  |  |  | 448  1699 |  |  |  |  |  |
| Info_news  Low use  High use |  |  |  | 293  1854 |  |  |  |  |  |
| Info_webdocs.ic  Low use  High use |  |  |  | 1224  924 |  |  |  |  |  |
| Info_healthports  Low use  High use |  |  |  | 1120  1030 |  |  |  |  |  |
| Country: Malaysia | | | | | | | | | |
| DHL_search | 3.09 | 0.49 | 0.017 | 861 | Binary logistic regression | 0.167 | 0.593 | 0.224 | 0.150 |
| DHL_content | 2.76 | 0.56 | 0.020 | 760 |  | 0.504 | 0.630 | 0.642 | 0.039 |
| DHL_reliability | 2.83 | 0.56 | 0.019 | 861 |  | 0.009 | 0.886 | 0.988 | 0.255 |
| DHL_relevance | 3.01 | 0.45 | 0.015 | 861 |  | 0.018 | 0.282 | 0.125 | 0.414 |
| DHL_privacy | 3.24 | 0.66 | 0.024 | 736 |  | 0.655 | 0.268 | 0.352 | 0.884 |
| Info_webpublic  Low use  High use |  |  |  | 355  501 |  |  |  |  |  |
| Info_news  Low use  High use |  |  |  | 149  705 |  |  |  |  |  |
| Info_webdocs.ic  Low use  High use |  |  |  | 531  323 |  |  |  |  |  |
| Info_healthports  Low use  High use |  |  |  | 259  597 |  |  |  |  |  |
| **Table 4** |  |  |  |  |  |  |  |  |  |
| ALL countries | Mean | SD | SE | N | Statistical method | Info_satisf (p-value) | |  |  |
| DHL_search | 3.01 | 0.51 | 0.017 | 4906 | Binary logistic regression | 0.000 |  |  |  |
| DHL_content | 2.76 | 0.60 | 0.009 | 4644 |  | 0.133 |  |  |  |
| DHL_reliability | 2.78 | 0.58 | 0.008 | 4900 |  | 0.022 |  |  |  |
| DHL_relevance | 3.00 | 0.48 | 0.007 | 4898 |  | 0.000 |  |  |  |
| DHL_privacy | 3.13 | 0.72 | 0.011 | 4504 |  | 0.083 |  |  |  |
| Info_Importance | 3.62 | 0.39 | 0.006 | 4893 |  | 0.001 |  |  |  |
| Info_satisf  Low  High |  |  |  | 1889  3001 |  |  |  |  |  |
| Country: China |  |  |  |  |  |  |  |  |  |
| DHL_search | 3.05 | 0.51 | 0.012 | 1884 | Binary logistic regression | 0.000 |  |  |  |
| DHL_content | 2.86 | 0.61 | 0.014 | 1799 |  | 0.357 |  |  |  |
| DHL_reliability | 2.67 | 0.57 | 0.013 | 1884 |  | 0.172 |  |  |  |
| DHL_relevance | 2.98 | 0.47 | 0.011 | 1883 |  | 0.000 |  |  |  |
| DHL_privacy | 3.16 | 0.78 | 0.019 | 1725 |  | 0.049 |  |  |  |
| Info_Importance | 3.43 | 0.43 | 0.010 | 1882 |  | 0.006 |  |  |  |
| Source1_searcheng  Low use  High use |  |  |  | 263  1620 |  | 0.271 |  |  |  |
| Source2_webpublic  Low use  High use |  |  |  | 751  1130 |  | 0.484 |  |  |  |
| Source3_wiki  Low use  High use |  |  |  | 712  1170 |  | 0.833 |  |  |  |
| Source4_socmed  Low use  High use |  |  |  | 236  1646 |  | 0.068 |  |  |  |
| Source5_youtube  Low use  High use |  |  |  | 696  1184 |  | 0.593 |  |  |  |
| Source6_healthblogs  Low use  High use |  |  |  | 1131  750 |  | 0.322 |  |  |  |
| Source7_onlinecoms  Low use  High use |  |  |  | 1178  702 |  | 0.175 |  |  |  |
| Source8_healthports  Low use  High use |  |  |  | 1189  690 |  | 0.009^**^ |  |  |  |
| Source9_webdoctors  Low use  High use |  |  |  | 1405  474 |  | 0.905 |  |  |  |
| Source10_newsports  Low use  High use |  |  |  | 439  1442 |  | 0.108 |  |  |  |
| Topic1_Currentspread |  |  |  | 1718 |  | 0.120 |  |  |  |
| Topic2_Transmission |  |  |  | 1542 |  | 0.414 |  |  |  |
| Topic3_Symptoms |  |  |  | 1590 |  | 0.191 |  |  |  |
| Topic4_Protection |  |  |  | 1383 |  | 0.207 |  |  |  |
| Topic5_Hygiene |  |  |  | 1111 |  | 0.323 |  |  |  |
| Topic6_Situationassess |  |  |  | 997 |  | 0.148 |  |  |  |
| Topic7_Restrictions |  |  |  | 1402 |  | 0.749 |  |  |  |
| Topic8_Socioecoconseq |  |  |  | 956 |  | 0.518 |  |  |  |
| Topic9_Stressmgt |  |  |  | 617 |  | 0.165 |  |  |  |
| Info_satisf  Low  High |  |  |  | 876  1005 |  | 0.120 |  |  |  |
| Country: Philippines | Mean | SD | SE | N | Statistical method | Info_satisf (p-value) | |  |  |
| DHL_search | 2.94 | 0.51 | 0.011 | 2161 | Binary logistic regression | 0.000 |  |  |  |
| DHL_content | 2.68 | 0.59 | 0.013 | 2085 |  | 0.896 |  |  |  |
| DHL_reliability | 2.86 | 0.59 | 0.013 | 2155 |  | 0.082 |  |  |  |
| DHL_relevance | 3.01 | 0.49 | 0.011 | 2154 |  | 0.013 |  |  |  |
| DHL_privacy | 3.06 | 0.67 | 0.015 | 2043 |  | 0.599 |  |  |  |
| Info_Importance | 3.80 | 0.25 | 0.005 | 2154 |  | 0.037 |  |  |  |
| Source1_searcheng  Low use  High use |  |  |  | 109  2049 |  | 0.123 |  |  |  |
| Source2_webpublic  Low use  High use |  |  |  | 448  1699 |  | 0.1430 |  |  |  |
| Source3_wiki  Low use  High use |  |  |  | 1071  1081 |  | 0.353 |  |  |  |
| Source4_socmed  Low use  High use |  |  |  | 174  1984 |  | 0.930 |  |  |  |
| Source5_youtube  Low use  High use |  |  |  | 481  1672 |  | 0.373 |  |  |  |
| Source6_healthblogs  Low use  High use |  |  |  | 923  1229 |  | 0.310 |  |  |  |
| Source7_onlinecoms  Low use  High use |  |  |  | 1315  831 |  | 0.128 |  |  |  |
| Source8_healthports  Low use  High use |  |  |  | 1120  1030 |  | 0.699 |  |  |  |
| Source9_webdoctors  Low use  High use |  |  |  | 1224  924 |  | 0.049 |  |  |  |
| Source10_newsports  Low use  High use |  |  |  | 293  1854 |  | 0.639 |  |  |  |
| Topic1_Currentspread |  |  |  | 1950 |  | 0.650 |  |  |  |
| Topic2_Transmission |  |  |  | 1327 |  | 0.822 |  |  |  |
| Topic3_Symptoms |  |  |  | 1838 |  | 0.349 |  |  |  |
| Topic4_Protection |  |  |  | 1544 |  | 0.758 |  |  |  |
| Topic5_Hygiene |  |  |  | 1563 |  | 0.051 |  |  |  |
| Topic6_Situationassess |  |  |  | 1253 |  | 0.440 |  |  |  |
| Topic7_Restrictions |  |  |  | 1053 |  | 0.125 |  |  |  |
| Topic8_Socioecoconseq |  |  |  | 1284 |  | 0.740 |  |  |  |
| Topic9_Stressmgt |  |  |  | 1039 |  | 0.158 |  |  |  |
| Info_satisf  Low  High |  |  |  | 748  1405 |  |  |  |  |  |
| Country: Malaysia | Mean | SD | SE | N | Statistical method | Info_satisf (p-value) | |  |  |
| DHL_search | 3.09 | 0.49 | 0.017 | 861 | Binary logistic regression | 0.006 |  |  |  |
| DHL_content | 2.76 | 0.56 | 0.020 | 760 |  | 0.038 |  |  |  |
| DHL_reliability | 2.83 | 0.56 | 0.019 | 861 |  | 0.826 |  |  |  |
| DHL_relevance | 3.01 | 0.45 | 0.015 | 861 |  | 0.000 |  |  |  |
| DHL_privacy | 3.24 | 0.66 | 0.024 | 736 |  | 0.756 |  |  |  |
| Info_Importance | 3.57 | 0.38 | 0.013 | 857 |  | 0.458 |  |  |  |
| Source1_searcheng  Low use  High use |  |  |  | 22  836 |  | 0.709 |  |  |  |
| Source2_webpublic  Low use  High use |  |  |  | 355  501 |  | 0.536 |  |  |  |
| Source3_wiki  Low use  High use |  |  |  | 403  451 |  | 0.519 |  |  |  |
| Source4_socmed  Low use  High use |  |  |  | 158  698 |  | 0.751 |  |  |  |
| Source5_youtube  Low use  High use |  |  |  | 261  591 |  | 0.168 |  |  |  |
| Source6_healthblogs  Low use  High use |  |  |  | 558  298 |  | 0.421 |  |  |  |
| Source7_onlinecoms  Low use  High use |  |  |  | 492  361 |  | 0.378 |  |  |  |
| Source8_healthports  Low use  High use |  |  |  | 259  597 |  | 0.546 |  |  |  |
| Source9_webdoctors  Low use  High use |  |  |  | 531  323 |  | 0.839 |  |  |  |
| Source10_newsports  Low use  High use |  |  |  | 149  705 |  | 0.010 |  |  |  |
| Topic1_Currentspread |  |  |  | 792 |  | 0.787 |  |  |  |
| Topic2_Transmission |  |  |  | 489 |  | 0.070 |  |  |  |
| Topic3_Symptoms |  |  |  | 671 |  | 0.176 |  |  |  |
| Topic4_Protection |  |  |  | 502 |  | 0.464 |  |  |  |
| Topic5_Hygiene |  |  |  | 480 |  | 0.857 |  |  |  |
| Topic6_Situationassess |  |  |  | 475 |  | 0.224 |  |  |  |
| Topic7_Restrictions |  |  |  | 606 |  | 0.685 |  |  |  |
| Topic8_Socioecoconseq |  |  |  | 445 |  | 0.144 |  |  |  |
| Topic9_Stressmgt |  |  |  | 254 |  | 0.456 |  |  |  |
| Info_satisfaction  Low  High |  |  |  | 265  591 |  | 0.787 |  |  |  |
